# Evaluation of the Antiviral Activity of Cuscuta Extracts Against HSV-1, HSV-2 (ACV-Resistant Strain), HCoV229E and SARS-CoV-2

**DOI:** 10.3390/ijms27188120

**Published:** 2026-09-12

**Authors:** Zornitsa Tileva, Petya Angelova, Anton Hinkov, Stoyan Shishkov, Lyuben Zagorchev, Denitsa Teofanova, Ivo Sirakov, Kalina Shishkova

**Affiliations:** 1 Laboratory of Virology, Faculty of Biology, University of Sofia “St. Kliment Ohridski”, 1164 Sofia, Bulgaria; ztileva@gmail.com (Z.T.); pjangelova@uni-sofia.bg (P.A.); ahinkov@biofac.uni-sofia.bg (A.H.); sshishkov@biofac.uni-sofia.bg (S.S.); 2 Centre of Competence “Sustainable Utilization of Bio-Resources and Waste of Medicinal and Aromatic Plants for Innovative Bioactive Products” (BIORESOURCES BG), 1000 Sofia, Bulgaria; 3 Department of Biochemistry, Faculty of Biology, Sofia University “St. Kliment Ohridski”, 1164 Sofia, Bulgaria; lzagorchev@biofac.uni-sofia.bg (L.Z.); teofanova@biofac.uni-sofia.bg (D.T.); 4 Department of Medical Microbiology “Corr. Mem. Prof. Ivan Mitov, MD, DMSc”, Faculty of Medicine, Medical University of Sofia, 1431 Sofia, Bulgaria; insirakov@gmail.com

**Keywords:** antiviral activity, viruses, plants extracts, inhibition, COVID-19, herpesviruses

## Abstract

The toxicological risk and development of resistance associated with synthetic drugs and supplements can be reduced by focusing efforts on medicinal plants that are of interest in pharmacognostic research to find new drugs or templates for the development of new bioactive compounds. The parasitic plant *Cuscuta* spp. has significant pharmacological activity at the extract level, but there is limited data on the bioactivity of its chemical compounds. In the present study, we aimed to investigate the antiviral potential of extracts obtained from different parts of two species, *C. campestris* and *C. epithymum*. The two species clearly differ in their flavonoid profiles, which could also suggest different antiviral activities of the extracts. We used methanol and aqueous extracts, MDBK, VERO E6 clone 76 and BGM cell lines, HSV-1, HSV-2 strain DD (acyclovir-resistant), HCoV-229E and SARS-CoV-2, maximum tolerable concentration, the MTT test, the plaque-forming method and the HPLS-MS analysis. It was found that the metabolic composition varies depending on the host plant. We hypothesize that differences in the composition of the extracts lead to differences in antiviral activity. Some of the tested methanol extracts showed a pronounced inhibitory effect, both on replication and on extracellular virions, in relation to the ACV-resistant strain of HSV type 2 and SARS-CoV-2 in comparison with the other viral models used by us. The strongest antiviral effect was shown by the methanol extract of *C. campestris* with host *Chenopodium* spp. against SARS-CoV-2, as the virus inhibition was about 100%, and the virucidal effect showed a decrease in the viral titer by more than 2.5 lg. Based on the results obtained, we assume possible antagonistic interaction of individual metabolites in the total methanol extracts. Extract C3, applied at the maximum tolerable concentration and one level lower, inhibits SARS-CoV-2 about 100 percent. SARS-CoV-2-infected cells preserved mitochondrial activity and relatively preserved lysosomal activity; this is also the reason why the MTT test, as well as neutral red staining, is not applicable to this viral model propagated in the VERO E6 cell line.

## 1. Introduction

Human herpes simplex viruses (HSV), belonging to the family *Herpesviridae*, subfamily *Alphaherpesvirinae*, are widespread human pathogens with a global seropositivity of 66%, comprising type 1 (HSV-1) and type 2 (HSV-2) [1]. Herpesvirus infections cause a range of diseases, including primary and recurrent mucosal infections (gingivostomatitis, herpes labialis and genital infections), neonatal and congenital infection, eczema herpeticum in patients with atopic dermatitis, and remain latent in neurons for the rest of life [1,2]. In rare cases, HSV infections can lead to encephalitis and meningitis [3]. After infecting sensory nerves, HSV-1 reaches the neuron bodies by retrograde axonal transport, where it establishes a lifelong latent infection in the trigeminal and dorsal root ganglia. Upon reactivation, the virus is transported anterogradely to peripheral epithelial cells, where in immunocompetent individuals it most often causes recurrent oral or labial lesions [4,5].

Coronaviruses belong to the *Coronaviridae* family and are common pathogens infecting a wide range of hosts, including humans [6]. COVID-19 (Coronavirus Disease 2019) was the largest pandemic observed in the 21st century, causing over 123.5 million reported cases and 2.7 million deaths worldwide [7]. Coronaviruses are enveloped viruses with a large, single-stranded positive-sense RNA genome. Four coronaviruses circulate primarily in the human population—two *alphacoronaviruses* (NL63 and 229E) and two *betacoronaviruses* (HKU1 and OC43). All four viruses are believed to be of zoonotic origin, with three of them (SARS-CoV, MERS-CoV and SARS-CoV-2) having infected humans after crossing the species barrier [6,8]. Coronaviruses, as RNA viruses, have a high mutation rate, which, in addition to leading to the emergence of new strains, allows them to adapt to a wide range of hosts [9,10]. Clinical signs and symptoms include respiratory, renal, neurological and enteric manifestations, as well as other forms of the disease [9]. One of the main factors determining the tropism of the virus and the ability to cross the interspecies barrier is the receptor-binding domain (RBD), a region located in the spike (S) protein. This region is responsible for binding to the host receptor, angiotensin I-converting enzyme 2 (ACE2). The virus is more easily adapted to new hosts by using cellular proteases, furin, and the ability to evade or exploit the host immune response. Amino acid substitutions or point mutations associated with the replacement of one amino acid with another amino acid in the RBD region can lead to a change in the specificity of receptor binding, which in turn leads to a change in the transmission routes and pathogenic potential of the virus. All of these changes can lead to a more severe course of the disease [10,11].

Despite the availability of certain antiviral drugs, for many viral infections there is no effective therapy in the first place due to emerging resistance caused by emerging mutations, toxicity and undesirable side effects [12,13,14].

At the onset of the pandemic, several antiviral agents were used to control SARS-CoV-2 infection [15,16,17,18]. Remdesivir is a nucleotide analogue that inhibits viral RNA-dependent RNA polymerase and was one of the first antiviral agents to receive emergency use authorization from the FDA (U.S. Food and Drug Administration) for the treatment of COVID-19. Monoclonal antibodies targeting the S-protein of the virus are also used, but they are not sufficiently effective against the relatively rapidly emerging new variants of the virus [18].

In RNA viruses, relatively high mutation rates contribute to the emergence of new subtypes, which in turn could lead to the emergence of resistance to current therapy [19]. On the other hand, many antiviral therapies exhibit undesirable side effects, limiting their use and efficacy [14]. Given the challenges faced by antiviral therapy, such as relatively high toxicity and a relatively rapid emergence of resistant mutants, it is necessary to look at other alternative options. Such an alternative option is medicinal plants, as they possess a set of diverse bioactive compounds, including secondary metabolites such as alkaloids, flavonoids and terpenes, which have a complex structure and chemical diversity [14,19]. It is this diversity and multicomponent composition of plant extracts, according to a number of author groups, that suggests possible attacks on various viral targets. Attacking different viral targets reduces the risk of resistance development, as there is a low probability that viruses will become simultaneously resistant to multiple secondary metabolites present in plant extracts [12,14,19].

*Cuscuta* is the single parasitic genus in the morning glory (*Convolvulaceae*) family and includes around 200 species of worldwide-distributed stem holoparasitic plants, called dodders [20]. The greatest diversity has been found in North and South America, and around 50 species have been established in the USA alone [21]. Several species are also found in Europe, either native or introduced, of which nine are naturally distributed in Bulgaria [22,23]. Due to their high content of bioactive compounds, *Cuscuta* spp. are also considered to be plants with medicinal potential. In addition to the species *Cuscuta chinensis* and *C. australis*, which are widely used in traditional Chinese medicine [24], over 15 other species are considered to be medicinally significant, including *C. campestris* and *C. epithymum*, which are also found in Bulgaria [22,23]. Plants of the genus *Cuscuta* contain a variety of secondary metabolites, including flavonoids and phenolic compounds, and the composition of plant extracts can vary depending on the species and host plant. The polyphenolic profile was proposed as a way to distinguish among different *Cuscuta* species, as the levels of different compounds within this group vary significantly between species [24,25]. Extracts of *Cuscuta* spp. have also been used in Bulgarian traditional medicine [26]. In the present study, we aimed to investigate the antiviral potential of extracts obtained from different parts of two species—*C. campestris* and *C. epithymum*. The two species clearly differ in their flavonoid profiles [27], which could also suggest different antiviral activity and antioxidant properties of the extracts [28]. Although the antiviral potential of *Cuscuta* species has been previously documented, the literature has largely neglected how the host plant modulates this activity, especially against emerging and drug-resistant pathogens. Since Cuscuta plants rely entirely on their host for nutrients and secondary metabolites, their antiviral efficacy can vary significantly depending on the host species. Given these facts, after examining six extracts from different parts of *C. campestris* and *C. epithymum* with different solvents and in view of the results obtained, we continued with methanol extracts of *C. campestris* collected from the same habitat but parasitizing two different hosts: *Polygonum aviculare* (C1) and *Chenopodium* spp. (C3). The extracts were selected to assess how host selection affects the composition of secondary metabolites and the antiviral potential of the plant against SARS-CoV-2, as well as against acyclovir-sensitive and acyclovir-resistant strains of herpes simplex virus (HSV).

## 2. Results

### 2.1. Flavonoid Profile of Aqueous and Methanol Extracts of Stem and Flower of C. campestris and C. epithymum

Although largely nonpolar, the recovery rate and stability of certain flavonoid compounds depend significantly on the type of solvent and its concentration [29]. It was found that in *C. campestris*, the recovery was higher at lower concentrations of alcoholic solvents, while in *C. epithymum* the opposite trend was observed—the highest recovery of polyphenols was achieved with concentrated ethanol and methanol [30]. A comparative analysis of the flavonoid profiles was performed between the two species based on the areas of the integrated peaks of the compounds from UV chromatograms.

Based on the results, 100% methanol extraction was used to further extract flavonoids from the collected samples, giving a high total polyphenol content in both species. Thirteen compounds were identified in the six methanol extracts of stem and flower of *C. campestris* and *C. epithymum*, of which 11 were flavonoid compounds and two were quinic acid derivatives. The flavonoid compounds detected were as follows: 1. *Kaempferol-3,7-O-diglucoside*, 2. *Quercetin-3-O-galactoside*, 3. *Quercetin-3-O-glucoside*, 4. *Kaempferol-3-O-glalactoside*, 5. *Astragalin*, 6. *Isorhamnetin-7-glucoside*, 7. *Isorhamnetin-3-O-glucoside*, 8. *quercetin*, 9. *flavone*, 10. *kaempferol*, 11. *isorhamnetin*. All ten flavonoids are members of the flavonol class. Compounds 1 and 5 differed dramatically in their UV spectra from the other eleven compounds. Based on the UV absorption spectrum, MS data, and literature search [27], compounds 1 and 5 were identified as *5-caffeoylquinic acid* and *dicaffeoylquinic acid*, respectively.

The 13 compounds identified, including *flavone*, *chlorogenic acid*, *hyperoside*, *isoquercitrin*, *kaempferol-3-O-glalactoside*, *quercetin*, *kaempferol-3,7-O-diglucoside* and *dicaffeoylquinic acid,* varied statistically significantly between the two species *C. campestris* and *C. epithymum*. The total polyphenol content was higher in *C. epithymum* compared with *C. campestris* [27]. The fact that the total polyphenol fraction in *C. epithymum* is higher does not necessarily mean higher antiviral activity. The effect of mixing flavonoids depends on whether they are present as free aglycones or as glycosides, which demonstrates that glycosylation can alter the nature and strength of interactions between polyphenolic components [31].

### 2.2. Flavonoid Profile of Methanol Extracts from Cuscuta campestris Stem—C1 and C3

**Figure 1:**
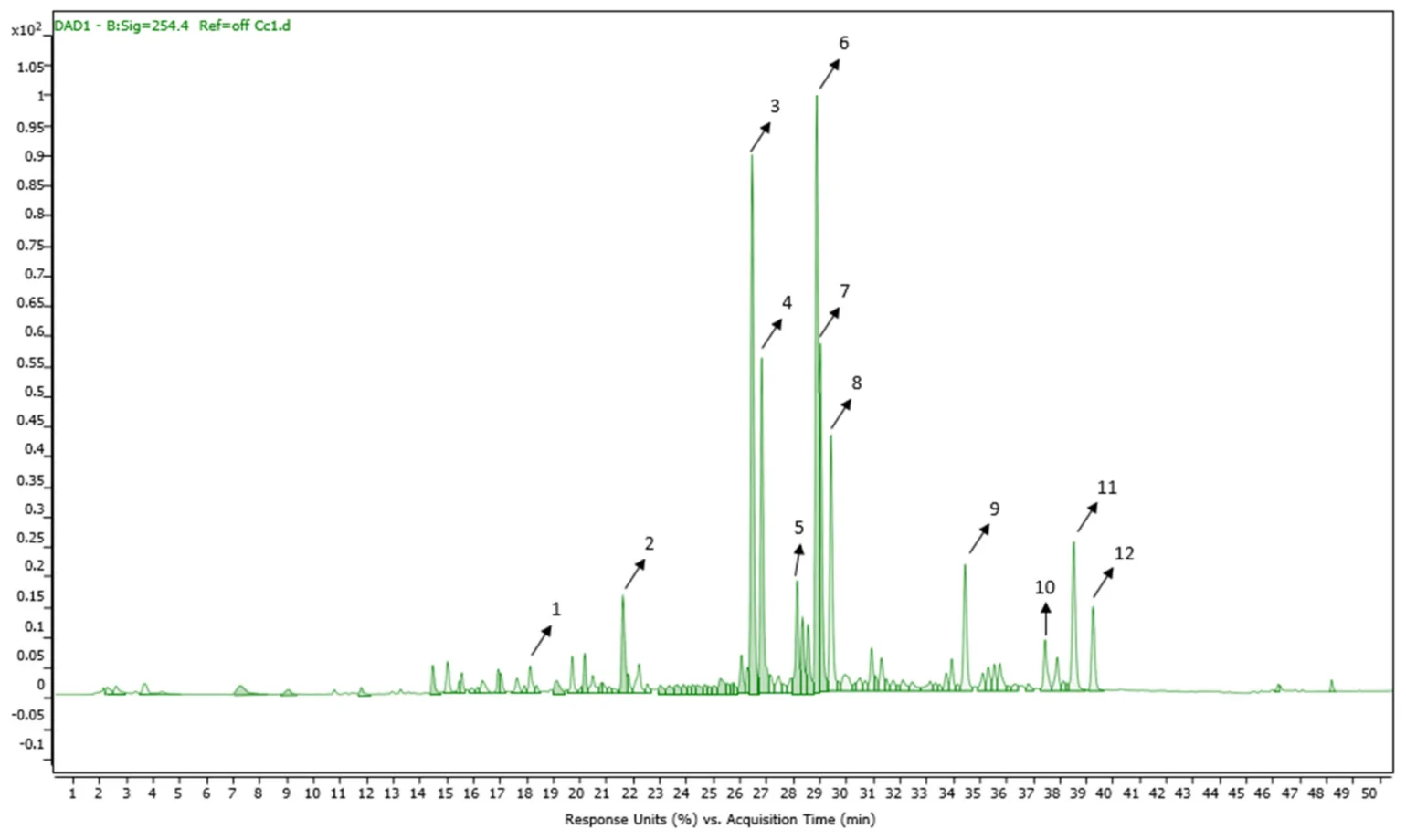
HPLC/MS chromatogram of the C1 extract at 190–500 nm.

**Figure 2:**
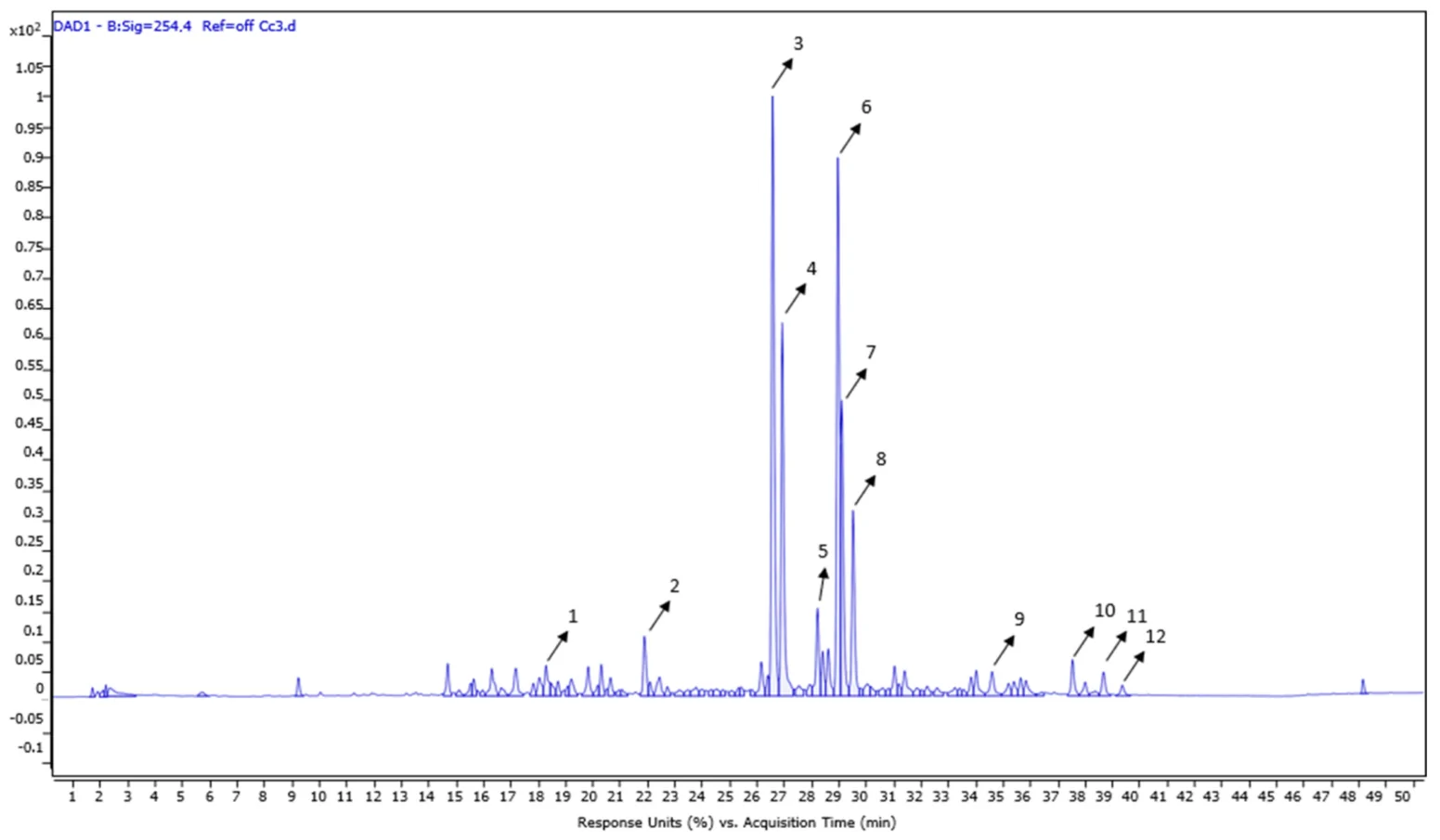
HPLC/MS chromatogram of the C3 extract at 190–500 nm.

The results of our previous study show that the content of polyphenols depends strongly not only on the environmental conditions in the specific locality [27], but also on the host, which is an inherent characteristic of parasitic plants. This is especially true for *Cuscuta* spp., considering that they are universalists compared to the more specialized root parasites of the family *Orobanchacea*. This is also one of the reasons for investigating the flavonoid composition of methanol extracts from the stem of *C. campestris* with the same habitat but different hosts, presented in Table 1, Figure 1 and Figure 2. We focused on the species *C. campestris* because of the more pronounced antiviral effect, described in Section 4.

As can be seen from Table 1, compounds were putatively identified, of which 11 are flavonoids belonging to the group of flavonols and their glycosides and one compound—*chlorogenic acid*—is a *phenolic acid.* A similar flavonoid profile was observed in both extracts (Figure 1 and Figure 2), but with visible differences in the aglycones *quercetin.* A total of 12 compounds were identified.

A comparative analysis of the HPLC chromatograms of extracts C1 and C3 was performed. A similar flavonoid profile was observed in both extracts (Figure 1 and Figure 2), but with visible differences in the aglycones *quercetin* (peak 9), *kaempferol* (peak 11) and *isorhamnetin* (peak 12). The LC/MS analysis of the extracts shows that compounds 9, 11 and 12 are “upregulated” in sample Cc1 (C1) compared with Cc3 (C3) (respectively, 2.29, 6.84 and 6.91 times more in sample Cc1).

### 2.3. Cytotoxicity of Methanolic and Aqueous Extracts of C. campestris and C. epithymum

**Figure 3:**
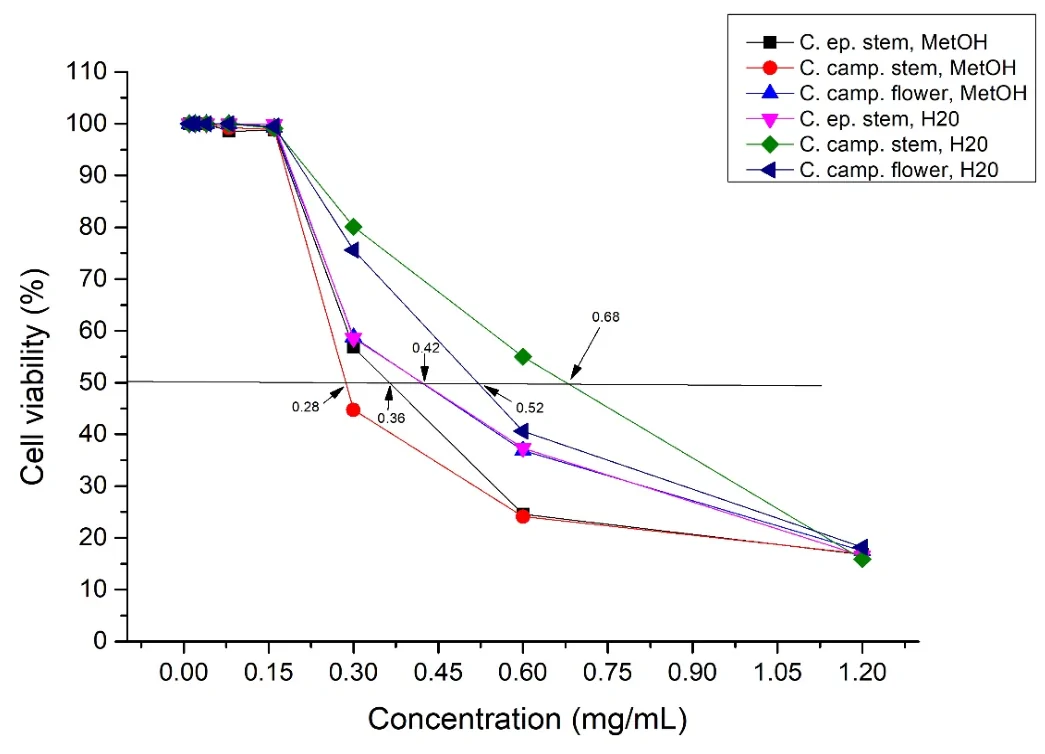
Survival of VERO E6 cell line cells exposed to methanol and aqueous extracts of *C. campestris* and *C. epithymum*. Arrows indicate the CC_50_ of the extracts, the value of which is determined from the constructed dose-response curve.

The extracts were applied at concentrations ranging from 1.2 to 0.01 mg/mL. The obtained values for MTC and CC_50_ for each of the extracts are presented in Table 2 and graphically depicted in Figure 3. The same MTC of 0.16 mg/mL was found for all the extracts studied. A difference was found in the obtained CC_50_ value, varying between 0.28 and 0.68 mg/mL. The lowest CC_50_ value was found for methanol extract of C. campestris stem (0.28 mg/mL), followed by methanol extract of *C. epithymum* stem (0.36 mg/mL). The same value for CC_50_ (0.42 mg/mL) was reported for the methanol extract of *C. campestris* flower and the aqueous extract of *C. epithymum* stem. When comparing the obtained data, it is evident that the methanol extract of *C. campestris* stem (0.28 mg/mL) has the greatest cytotoxicity, while the aqueous extract of *C. campestris* stem (0.68 mg/mL) exhibits the lowest cytotoxicity to VERO E6 cells. The Figure 3 shows the relationship between the applied concentration of the extracts and the percentage of cell protection.

### 2.4. Inhibition of Replication of HSV 1 (F), HSV 2 (DD) and HCoV229E in the Presence of the Tested Extracts

The inhibition of the replication cycle of HSV-1 strain F, the acyclovir-resistant HSV-2 strain DD and human coronavirus 229E was evaluated following treatment of cells infected with the respective viral models with the tested substances. The extracts were applied at the MTC and several concentrations below it (concentrations ranged from 0.16 to 0.01 mg/mL).

The antiviral potential of the tested extracts on human coronavirus 229E was assessed by an MTT assay. The experiment was reported at 72 h, but the results were not included due to the fact that % protection did not reach 50% (the highest percentage protection was about 20 when treated with methanol extract of *C. campestris* stem), and the virus was not used for further studies. The results of the antiviral effect for this virus model are attached as a Supplementary File (Supplementary Figure S1).

**Figure 4:**
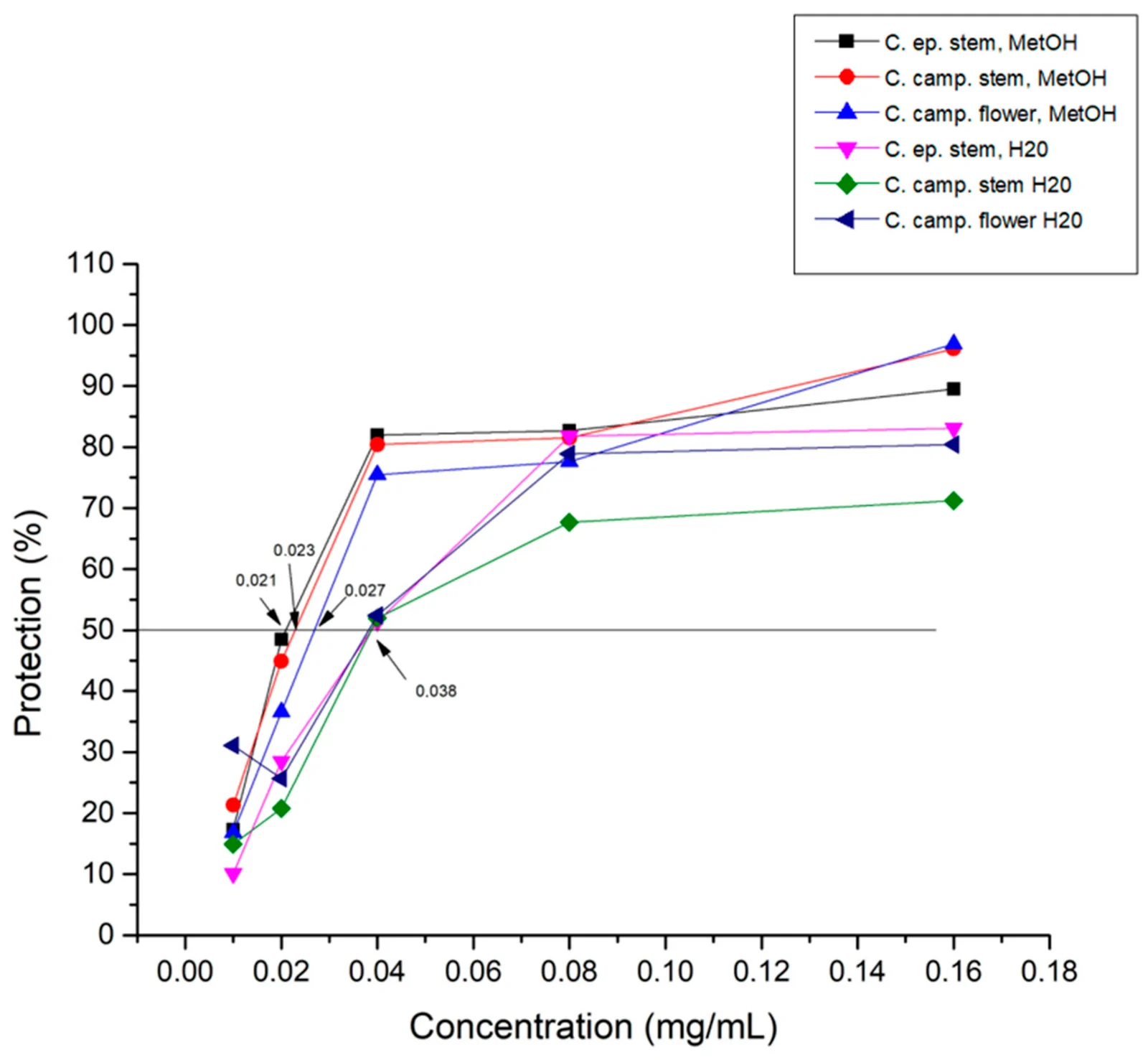
Antiviral activity of the tested extracts against replication of HSV-1 strain F.

**Figure 5:**
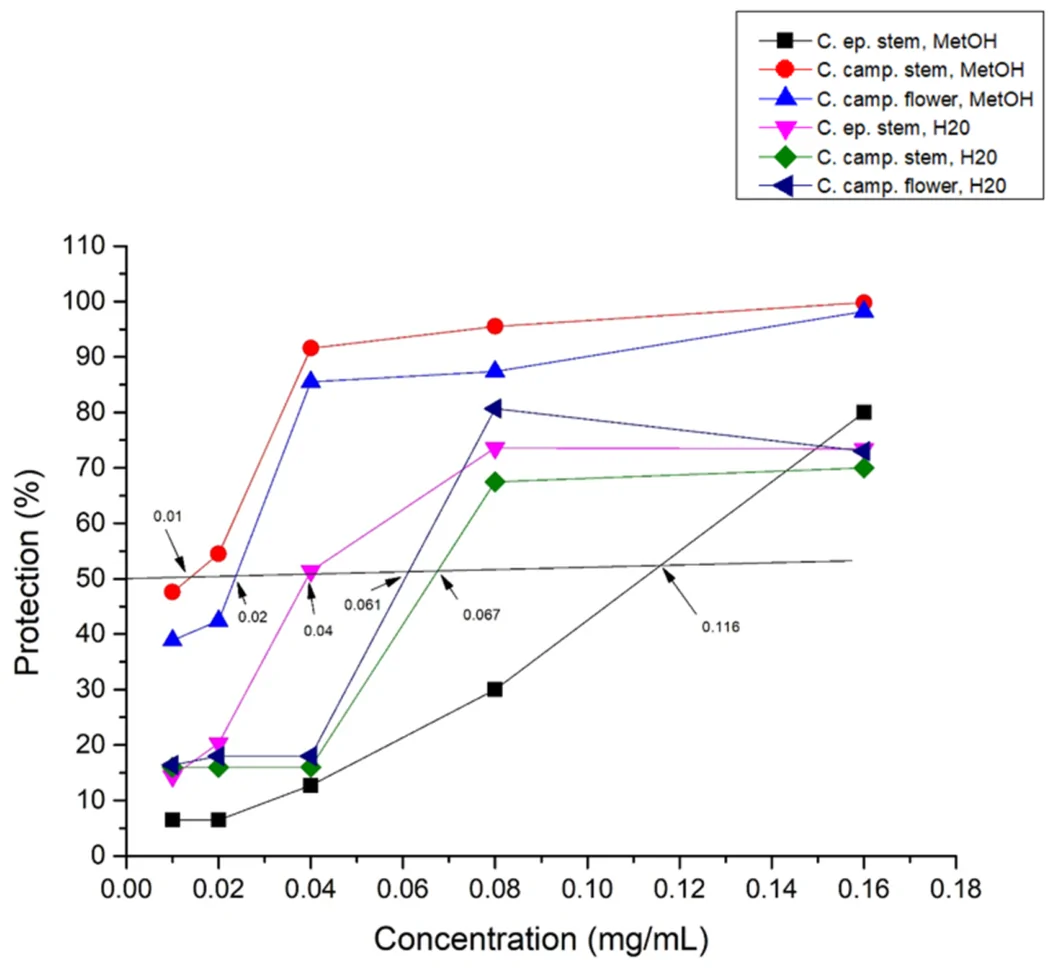
Antiviral activity of the tested extracts against the replication of HSV 2 strain DD (acyclovir-resistant). Compared to acyclovir, none of the extracts reached this SI. We find the result regarding the resistant herpes strain, against which acyclovir is ineffective, particularly valuable.

Figure 4 and Figure 5 graphically depict the percentage protection of cells infected with the respective virus and their treatment with each of the extracts. IC_50_ (inhibitory concentration 50) and SI (selective index) were calculated. Table 2 presents the results of the toxicity and antiviral activity against HSV 1 and 2 of the extracts.

ACV was used as a control preparation for HSV 1. The MTC determined for him was 0.0312 mg/mL and the CC_50_ was 0.341 mg/mL. The IC_50_ and SI were calculated and are as follows: 0.00121 mg/mL for IC50 and 277.23 for SI.

Inhibitory concentration 50 (IC_50_) represents the concentration of the test substance at which 50% of viral replication is inhibited and is determined graphically from the dose-response curve (Figure 4 and Figure 5). The selectivity index (SI) reflects the ratio of toxicity to antiviral effect of the substances. The obtained values for IC_50_ ranged from 0.021 to 0.038 mg/mL for HSV 1 (F) and from 0.01 to 0.116 mg/mL for HSV 2 (DD). The lowest value of IC_50_ against HSV 1 was found for the methanol extract of the stem of *C. epithymum* (0.021 mg/mL), followed by the methanol extract of the stem (0.023 mg/mL) and flower (0.027 mg/mL) of *C. campestris*. The three highest order selective index values, against HSV 1, were calculated for the aqueous extract of *C. campestris* stem, the methanol extract of *C. epithymum* stem and the methanol extract of *C. campestris* stem, respectively, 17.8, 17 and 15.5 (Table 2). Each of the experiments was repeated 3 to 5 times, and the trend was *p* < 0.05.

Figure 4 and Figure 5 show the relationship between the applied concentration of the extracts and the percentage of protection of cells infected with the respective virus.

The highest percentage of inhibition on HSV 1 (F) replication was achieved when infected cells were treated with methanol extracts of *C. campestris* flowers and stems, over 96%.

The antiviral potential of the studied extracts was also evaluated against HSV-2 strain DD. The obtained results for IC_50_ and SI are presented in Table 2 and graphically depicted in Figure 5.

The highest antiviral activity was found in the methanol extract of the stem of *C. campestris* with the lowest value of IC_50_ (0.01 mg/mL) and the highest calculated selectivity index (SI = 28). The least pronounced antiviral effect was found in the methanol extract of the stem of *C. epithymum* with the highest value of IC_50_ (0.116 mg/mL) and a low selectivity index (SI = 3.1).

The highest percentage of inhibition of the replication of the acyclovir-resistant strain was found when treating infected cells with the methanol extract of *C. campestris* stems, about 100%.

The difference in the action of the extracts on the two strains of herpes virus is impressive. We find the result of the strong antiviral effect of the methanol extract of *C. campestris* stems on the acyclovir-resistant strain particularly valuable. It is probably an effect on DNA polymerase, without requiring phosphorylation, since the strain is thymidine kinase negative. Although HSV-1 and HSV-2 are closely related viruses, there are amino acid differences between their enzymes and regulatory proteins. If the flavonoid binds directly to a viral protein (e.g., a protein involved in early replication), these differences may result in a higher affinity for HSV-2, which we find particularly valuable in our study. This is also the reason why we focus in the following experiments on two methanol extracts of *C. campestris* stems (C1, C3), but with different hosts of the parasitic plants, described in the Section 4.

### 2.5. Cytotoxicity and Antiviral Activity Against HSV-2 (DD) and SARS-CoV-2 of C1 and C3

The cell viability of two cell lines—MDBK and VERO E6—was evaluated in response to two methanol extracts of *Cuscuta campestris* stems—C1 and C3—but with different hosts. The results were recorded at 72 h, both microscopically and by MTT assay. The plant extracts were applied at concentrations from 1.2 mg/mL to 0.01 mg/mL. The determined MTC and CC_50_ are presented in Table 3.

The obtained values for MTC were the same for the two lines, as well as for the two extracts—0.16 mg/mL. Differences were noted in the graphic determination of CC_50_. The lowest recorded value of CC_50_ was for VERO E6 cells—0.296 mg/mL—and the highest for MDBK. From the obtained results, we see that the methanol extracts of *C. campestris* stems have the highest toxicity on VERO E6 cells and the lowest on MDBK cells.

Our team cultured human coronavirus 229E in MDBK cell culture, which had a clear cytopathic effect. In our attempts to culture SARS-CoV-2, which belongs to the genus Betacoronavirus, in the same cell line, there was no cytopathic effect at 48 h [32]. The BGM cell line was also used due to the fact that SARS-CoV-2 was isolated from a patient and the goal was to check to which cell lines it would adapt better and would exhibit a more distinct and specific cytopathic effect that would be preserved in the series of passages. In this cell line, a slight cytopathic effect appeared at the first and second passages, but after the third passage, the effect was lost.

**Figure 6:**
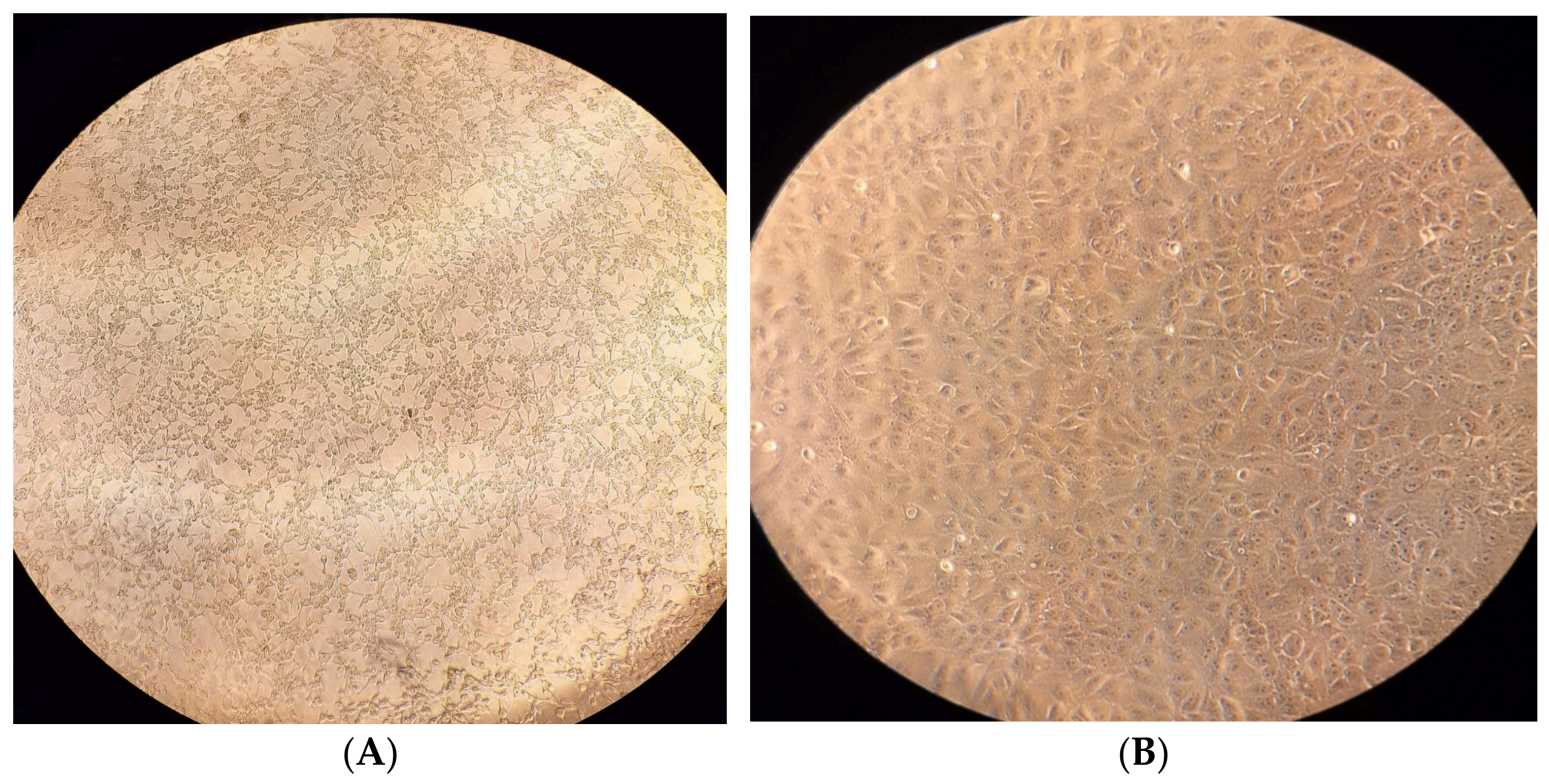
Cytopathic effect induced by SARS-CoV-2 in the VERO E6 cell line: (**A**) at 48 h; (**B**) cells from the VERO E6 cell line—uninfected.

In the VERO cell line, the virus exhibited a clear cytopathic effect at 48 h. When the virus was passaged in VERO cells, the cytopathic effect was evident at every passage. The cytopathic effect at 48 h of SARS-CoV-2 is presented in Figure 6.

Experiments with C1 and C3 assessed the replication of HSV-2 strain DD and SARS-CoV-2 by an MTT assay. VERO E6 cells were used, as they proved to be the most permissive for the isolated coronavirus. For the studies with HSV-2 strain DD, the MDBK cell line was used. The extracts were applied at the MTC previously determined and several lower than it (0.16 to 0.01 mg/mL). IC_50_ and SI were calculated and are presented in Table 4.

**Figure 7:**
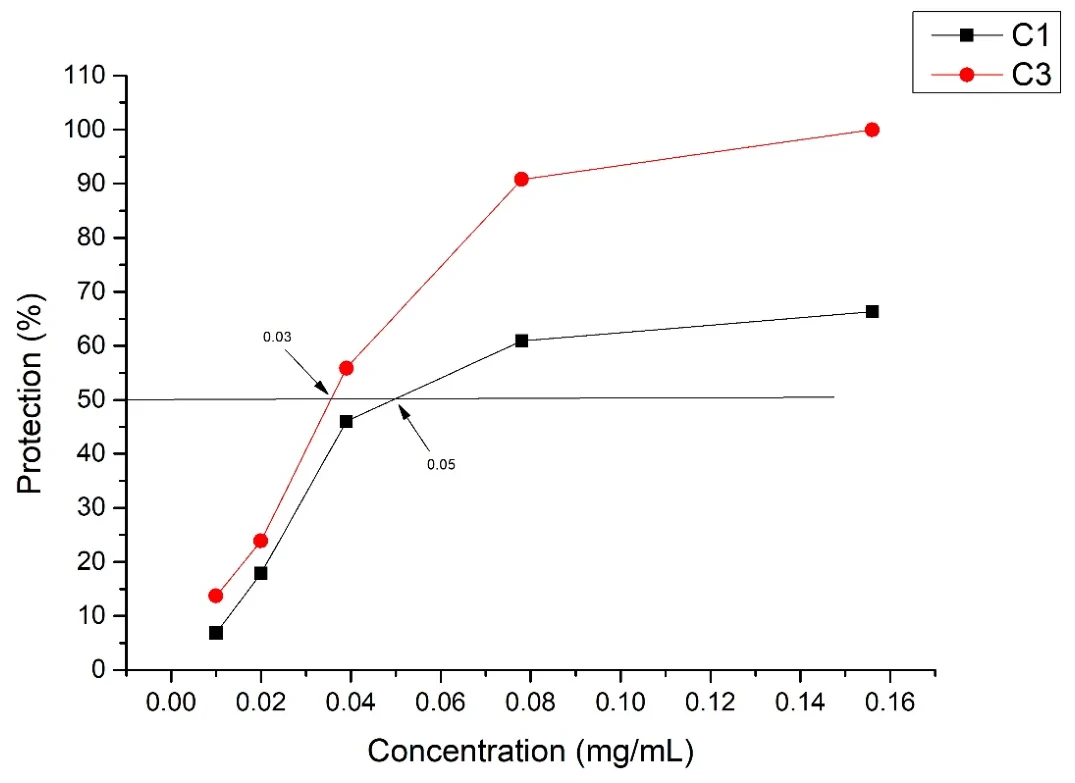
Antiviral activity of C1 and C3 on the replication of HSV-2 strain DD. Figure 7 shows the relationship between the applied concentration of the extracts and the percentage of protection of cells infected with the respective virus. The IC_50_ is indicated by arrows.

The tested extracts showed different antiviral activities. The IC_50_ values were determined from the dose-response curve and are graphically depicted in Figure 7. A higher IC_50_ value was determined for the C1 extract (0.05 mg/mL) with a selectivity index SI = 7.3. However, better antiviral activity was shown by the C3 extract with an IC_50_ of 0.03 mg/mL, a higher selectivity index (SI = 12) and a percentage of virus inhibition of 100% when the extract was applied at the MTC.

These findings suggest that mutations that confer resistance to acyclovir may reduce viral fitness. Such a virus may compensate by becoming more dependent on cellular signaling pathways or other life cycle steps that are inhibited by flavonoids.

The influence of the same extracts (C1 and C3) on the replication of SARS-CoV-2 was assessed by an MTT assay. The results were reported at 72 h, but there was no difference between the viral and cellular control values. The same experiment was also reported microscopically, where an almost complete and clearly expressed CPE was observed in the viral control (Supplementary Figure S2). This leads to the assumption that, in addition to the fact that the virus is not lytic, which is also visible microscopically and has been proven in our previous studies [32], it does not affect the mitochondrial activity of the cells, i.e., the infected cells have preserved mitochondrial activity. This led us to use another method for determining antiviral activity—the neutral red vitality test. An extremely small difference was found between the viral and cellular controls, again with a clearly expressed, microscopically reported cytopathic effect of the virus. The observed and reported spectrophotometric results show that the infected cells have relatively preserved lysosomal activity in addition to mitochondrial activity. These results showed us that the isolated virus is certainly not lytic and is suitable for reporting antiviral activity by the plaque-forming method—suitable for non-lytic viruses. Before proceeding with the plaque-forming method, we determined the antiviral activity of the extracts using the Reed and Muench method for determining viral titer [33], described in Section 4. The viral titer was reported at 48 h. When applying the C1 extract at the MTC (0.16 mg/mL), a decrease in the viral titer of more than 4 lg compared to the viral control was found, which corresponds to more than 99.99% inhibition of the virus. When treating the infected wells with a concentration of 0.08 mg/mL of C1, the decrease in the titer in the sample, compared to the control, was insignificant. When treated with the lower concentrations, no difference was observed between the samples and the control wells. When applying the C3 extract, better antiviral activity was found. When treating the samples with 0.16 and 0.08 mg/mL, no virus was detected in the samples. When applying the extract at a concentration of 0.04 mg/mL, the viral titer in the sample was over 2 lg lower compared to the control.

### 2.6. Determination of Antiviral Activity by Plaque-Forming Method of Methanol Extracts C1 and C3 Against SARS-CoV-2

**Figure 8:**
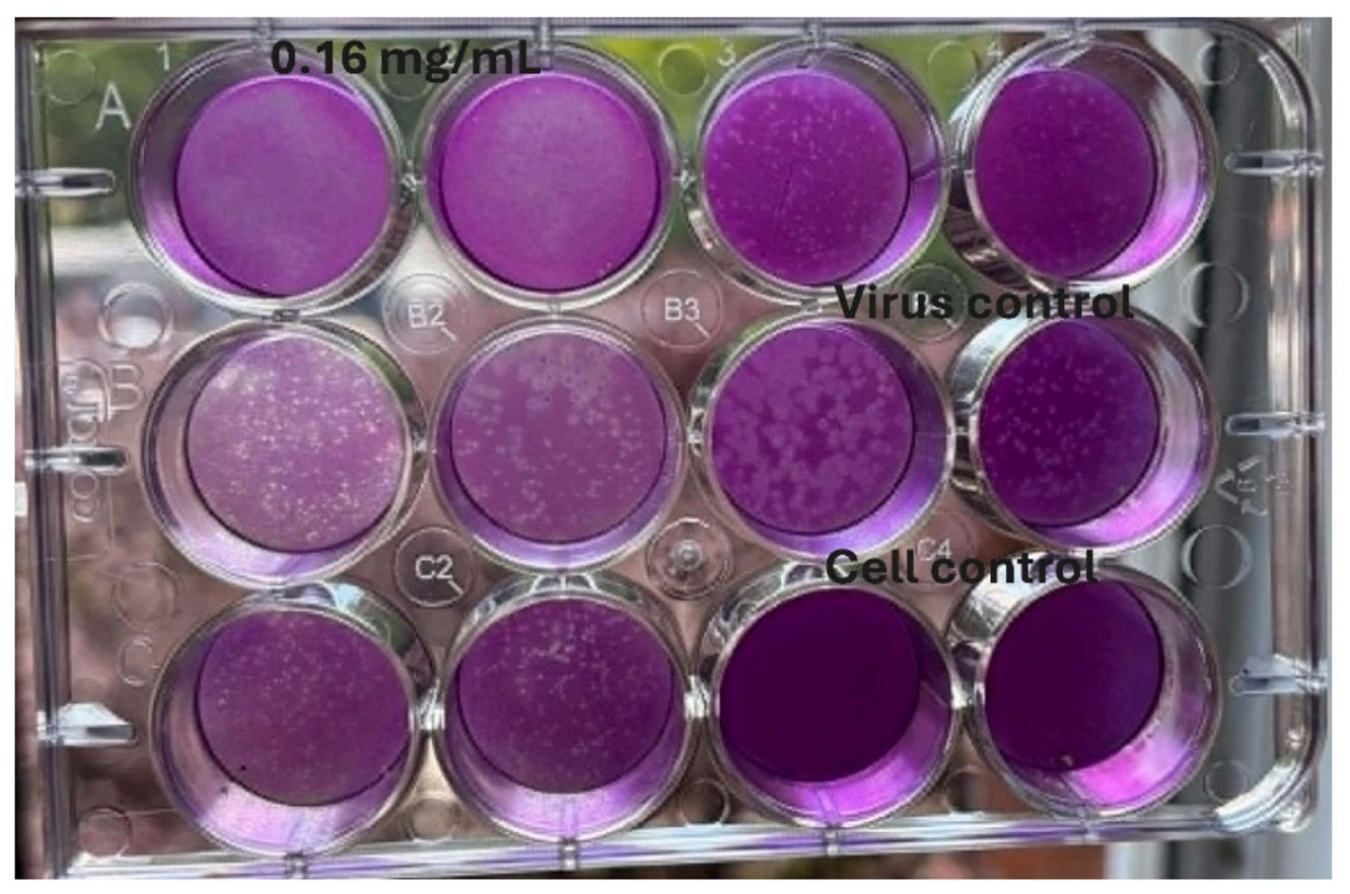
Inhibitory activity of the C1 methanol extract on plaque formation by SARS-CoV-2. Legend: Wells-A1 and A2—cells infected with the virus and treated with C1 extract at a concentration of 0.16 mg/mL. B1 and B2—cells infected with the virus and treated with C1 extract at a concentration of 0.08 mg/mL. C1 and A3—cells infected with the virus and treated with C1 extract at a concentration of 0.04 mg/mL. C2 and A4—cells infected with the virus and treated with C1 extract at a concentration of 0.02 mg/mL. B3 and B4—cells infected with the virus and untreated with extract. C3 and C4—cells uninfected with the virus and untreated with extract.

The extracts were applied at the MTC and several lower concentrations (0.16 to 0.02 mg/mL). A 1 × 10^−4^ dilution of the virus suspension was used as the viral control. The results were read at 72 h. When the C1 extract was applied at the MTC (0.16 mg/mL), no plaques were observed, indicating 100% inhibition of plaque formation (Figure 8). When treated with the second concentration below the MTC (0.08 mg/mL), a slight decrease in the number of plaques was observed compared to the viral control. At the following concentrations, no significant difference was observed compared to the viral control.

**Figure 9:**
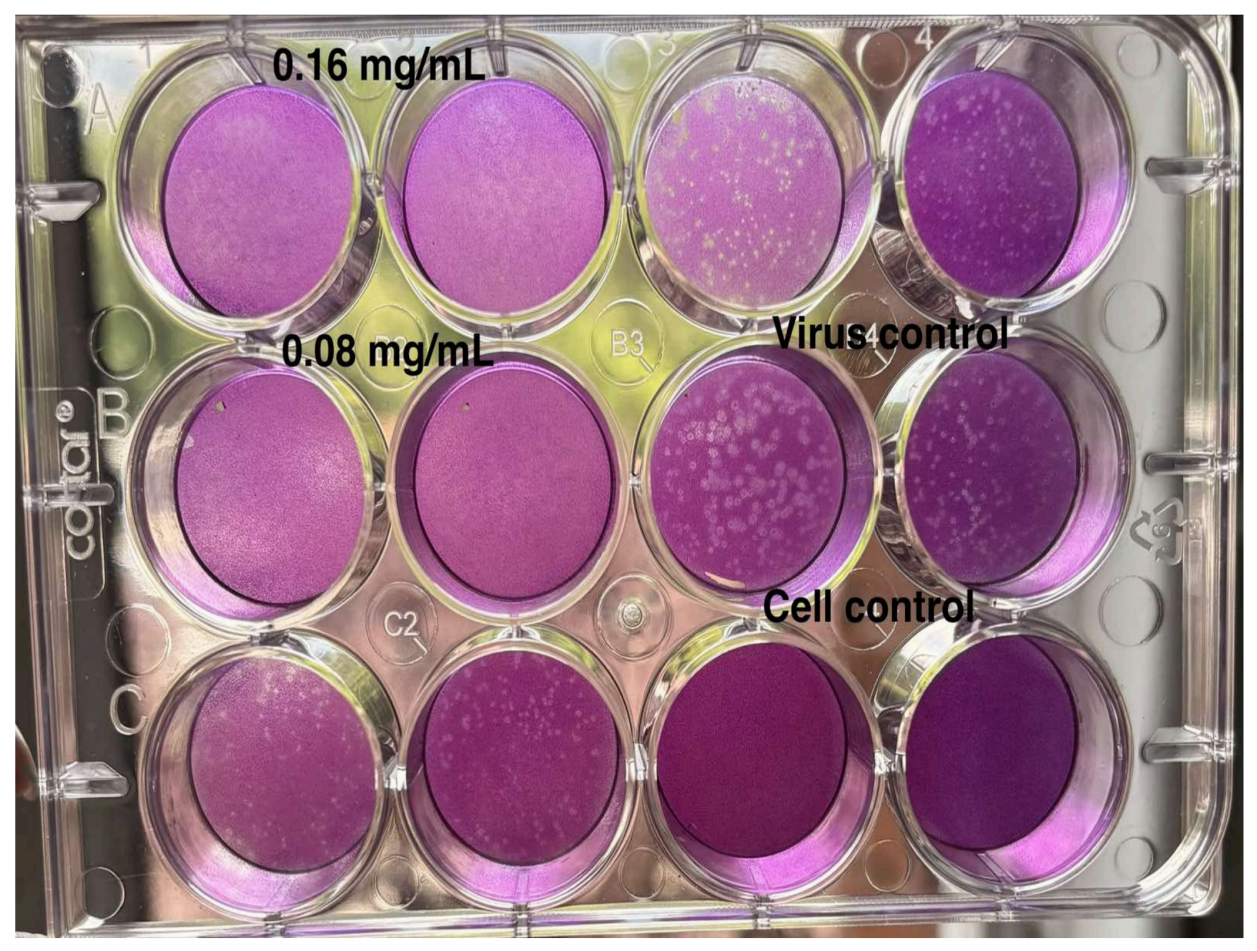
Antiviral activity of C3 against plaque formation by SARS-CoV-2. Legend: Wells-A1 and A2—cells infected with virus and treated with C3 extract at a concentration of 0.16 mg/mL. B1 and B2—cells infected with the virus and treated with C3 extract at a concentration of 0.08 mg/mL. C1 and C2—cells infected with the virus and treated with C3 extract at a concentration of 0.04 mg/mL. A3 and A4—cells infected with the virus and treated with C1 extract at a concentration of 0.02 mg/mL. B3 and B4—cells infected with the virus and untreated with extract. C3 and C4—cells uninfected with virus and untreated with extract.

The C3 extract showed better antiviral activity against SARS-CoV-2 compared to the C1 extract (Figure 9). At the MTC concentration and the next lower concentration (0.016 mg/mL and 0.08 mg/mL), the detection of plaques formed by the virus was below the detection limit. At the concentration of 0.04 mg/mL, the number of plaques was lower compared to the control. The summarized results are presented in Table 5.

### 2.7. Effect of C1 and C3 Methanol Extracts on Extracellular Virions of HSV-2 Strain DD and SARS-CoV-2

**Figure 10:**
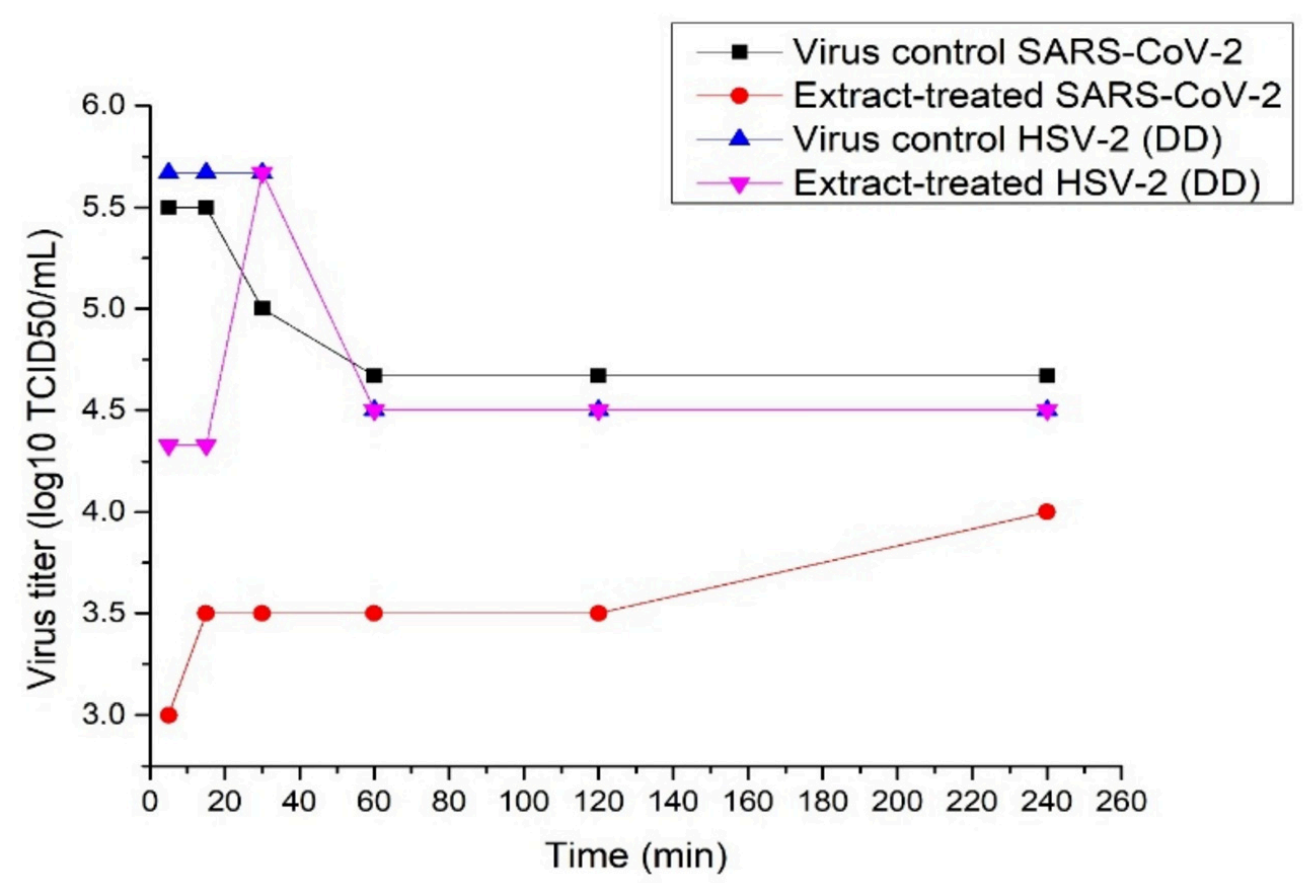
Virucidal effect of C3 on the extracellular forms of SARS-CoV-2 and HSV-2 strain DD.

When applying the C1 extract during the first 15 min, the viral titer of the sample decreased by 0.5 lg, which corresponds to a 67% inhibition of virions compared to the viral control. During the following time intervals, there was no difference in viral titers between the sample and the viral control, so the results were not included, and we continued by studying the C3 extract. The results of the virucidal effect of the C3 extract are summarized in Table 6 and graphically depicted in Figure 10.

The results presented in Table 6 show a clearly pronounced virucidal effect in the treatment of SARS-CoV-2. A significant virucidal effect was observed at 5 and 15 min of treatment, with differences of 2.5 and 2 lg between the titer of the sample and the control, respectively, which corresponds to about 99.9% inhibition of extracellular virions. With increasing time intervals, the differences in the titer of the sample and the control were about 1 lg (90% inhibition). With HSV-2 strain DD, a difference in the titer between the sample and the control was recorded only during the first 15 min, with the viral titer of the sample decreasing by just over 1 lg (90% inhibition). With increasing time intervals, no difference was found between the titer of the sample and the viral control. It can be concluded that, in general, the methanol extract of the stem of *C. campestris* exhibits higher activity both in terms of replication and against extracellular forms of SARS. An important result from a practical point of view, given that drugs for the treatment of resistant strains of HSV are very limited, is the relatively high SI value of C3 in relation to the ACV-resistant strain of HSV-2, considering that according to literature data, drugs with an SI above 10 are considered promising. Another significant result is the significant antiviral effect both on replication and on extracellular virions of SARS-CoV-2.

## 3. Discussion

Medicinal plants have a multicomponent and diverse composition, which suggests that they attack several viral targets. The presence of a cocktail of secondary metabolites reduces the risk of resistance development [12,14,19].

The toxicological risk and development of resistance associated with synthetic drugs and supplements can be reduced by focusing efforts on medicinal plants that are of interest in pharmacognostic research to find new drugs or templates for the development of new bioactive compounds. Plant-based drugs serve as a potential source of new biologically active agents whose safety and efficacy need to be validated. The parasitic plant *Cuscuta* spp. has significant pharmacological activity at the extract level, and there is limited data on the bioactivity of its chemical compounds. *Cuscuta campestris* and *Cuscuta epithymum* are used in traditional medicine, and in recent years there has been increasing interest in them due to their rich content of secondary metabolites [34,35]. The biological activities of *Cuscuta* spp. extracts include antioxidant, antimicrobial, anti-inflammatory and cytotoxic effects, probably due to the high content of phenolic compounds and flavonoids [34,35,36]. Phytochemical analyses using HPLC-MS have identified a number of flavonoid glycosides and phenolic acids in both species, including *hyperoside*, *isoquercitrin*, *quercetin*, *kaempferol* and their derivatives. Their composition has been found to vary depending on the host plant [30]. This area is poorly studied by researchers, so it is an interesting topic in the field of scientific research.

Based on our quantitative results, the 13 identified compounds varied statistically significantly between the two species *C. campestris* and *C. epithymum*, with the polyphenol content being higher in *C. epithymum*. When performing a comparative analysis by HPLC-MS of the two methanol extracts of *C. campestris* stems from the same habitat but different hosts—C1 with host *Polygonum aviculare* and C3 with host *Chenopodium* spp.—a similar flavonoid profile was obtained, with differences in the aglycones *quercetin*, *kaempferol* and *isorhamnetin*. These compounds were in higher abundance in sample C1 compared to sample C3. These differences in flavonoid profiles and in the amounts of secondary metabolites are most likely due to differences in hosts, which we assume is also the reason why the C3 extract exhibits higher antiviral activity. This proves our scientific hypothesis that host choice directly changes the pharmacological profile of the parasite.

However, when monitoring the antiviral activity of the two methanol extracts, we found that the C3 extract had significantly better antiviral activity against HSV-2 strain DD than the C1 extract. When determining antiviral activity by the plaque-forming method against SARS-CoV-2, significantly better activity of the C3 extract was again found, showing inhibition of plaque formation below the detection limit, at the MTC and the next lower concentration, while the C1 extract showed about 100% inhibition only at the MTC concentration, i.e., the plaques were below the detection limit.

The observed differences may be consistent with interactions between polyphenolic constituents, while emphasizing that specific combination experiments are needed to establish antagonism or synergy. All of the above and the analysis of the results obtained for the antiviral activity lead us to the assumption that the main significant antiviral activity of C3 is due to the glycosides, which are probably in antagonistic interactions with the larger amounts of aglycones in the C1 extract. The glycosides of flavonoid aglycones have similar viral targets to those of aglycones, but their affinities and mechanisms of action differ. For quercetin glycosides, the data on inhibition of the early stages of viral infection with respect to HSV-2, as well as the penetration of the virus, are best documented. This is probably due to the pronounced virucidal effect of the C3 extract—a decrease in the viral titer by about 1.5 lg already at the 5th minute. Another interesting result, in our opinion, is the lack of activity of the extracts against HCoV229E and the clear activity against SARS-CoV-2. The C3 extract showed better antiviral activity against SARS-CoV-2 compared to the C1 extract (Figure 9). At the MTC concentration and the next lower concentration (0.016 mg/mL and 0.08 mg/mL), the detection of plaques formed by the virus was below the detection limit. At the concentration of 0.04 mg/mL, the number of plaques was lower compared to the control.

We assume that these differences between the HPLC-MS analysis and the antiviral activity are due to possible antagonistic interactions between polyphenols and phenolic glycosides, as suggested by a number of authors [31]. The two extracts have similar overall flavonoid profiles, but differ significantly in the abundance of *quercetin, kaempferol* and *isorhamnetin*, with these aglycones being more abundant in C1.

Furthermore, the observed antiviral activity was stronger for C3, despite the higher abundance of these aglycones in C1, leading us to propose a possible explanation involving interactions between different classes of metabolites.

The observed differences may be consistent with interactions between polyphenolic constituents, while emphasizing that specific combination experiments are needed to establish antagonism or synergy. Based on our results showing relatively better activity of the C3 extract compared to the C1 extract, the obtained results for the flavonoid profile and the findings of other researchers, we hypothesize possible mechanisms of action. The clarification of the exact viral target and mechanism of action will be studied in further experiments.

In a study by Baranowska et al. [31], when comparing the aglycones quercetin and naringenin with their glycosides, it was found that the combination of flavonoids and their glycosides can change the electrochemical properties of flavonoids, leading to behavior that is different from that of the individual compounds. The effect of mixing flavonoids depends on whether they are present as free aglycones or as glycosides, which proves that glycosylation can change the nature and strength of the interaction between polyphenolic components [31]. Other authors found that the interaction between the aglycone *quercetin* and its glycoside *rutin* results in an antagonistic interaction, which is explained by a possible attraction between the glycosidic group and functional substituents of the molecules. The authors suggest that such an interaction may limit the accessibility of reactive hydroxyl groups. Jakobek et al. [37] noted that polyphenols can interact with carbohydrates, lipids and proteins, and these interactions can alter the activity and accessibility of phenolic compounds. In addition, hydrogen bonds, hydrophobic interactions and the formation of non-covalent complexes with plant components can limit the free fraction of some polyphenols and thus result in a lower effect than expected based on the chemical content [37]. Xiao et.al. showed that when a sugar residue is attached to flavonoids, their solubility, lipophilicity, absorption, metabolism and biological behavior are changed compared to their corresponding aglycones [38].

Although HSV-1 and HSV-2 are closely related viruses, there are amino acid differences between their enzymes and regulatory proteins. If the flavonoid is thought to bind directly to a viral protein (e.g., a protein involved in early replication), these differences may result in a higher affinity for HSV-2, which we find particularly valuable in our study. On the other hand, mutations that confer resistance to acyclovir may reduce viral fitness and indirectly increase its sensitivity to flavones or their glycosides. Such a virus may compensate by becoming more dependent on cellular signaling pathways or other life cycle steps that are inhibited by flavonoids. As a result, the resistant strain becomes relatively more sensitive to compounds with a different mechanism of action. The higher activity of the extracts against the acyclovir-resistant strain of HSV-2 is likely due to a mechanism of action different from that of acyclovir. While acyclovir requires activation by the viral thymidine kinase, flavonoids or their glycosides can act on the early stages of viral infection, inhibit the expression of viral alpha genes or modulate cellular signaling pathways such as NF-κB (nuclear factor kappa-light-chain-enhancer of activated B cells signaling pathway), MAPK (mitogen-activated protein kinase signaling pathway) and PI3K/Akt (Phosphoinositide 3-Kinase/Protein Kinase B (Akt) signaling pathway). All mechanisms of action suggested are hypothetical, supported by studies by other authors, and additional studies are needed to prove the mechanism of action.

One of the best studied quercetin glycosides against coronaviruses is *Quercetin-3-O-galactoside* [39,40]. It has been shown to bind to the catalytic pocket of SARS-CoV-2 3CLpro (major protease), which is highly conserved, and inhibition of 3CLpro is the main proposed mechanism of antiviral action. Another proposed mechanism of action is inhibition of PLpro. The fact is that this glycoside has a major target in influenza viruses, the RNA polymerase. It is not excluded that in coronaviruses, RdRp is also a target [39].

Although these enzymes are functionally similar, there are structural differences between HCoV-229E and SARS-CoV-2 in their active sites [41]. Since the extract contains several flavonoids, as well as their glycosides, it is possible that they have a higher affinity for the SARS-CoV-2 enzymes. Another possible explanation for this result is that the two viruses have a different mechanisms of replication and interaction with the cell, namely SARS-CoV-2 more strongly induces NF-κB, MAPK and oxidative stress, and if the extracts act by modulating these cellular processes, this may explain the stronger effect on SARS-CoV-2 than on HCoV-229E [42]. Since C3 was also reported to have a strong virucidal effect against SARS-2, we assume that the targets are either viral targeting proteins or viral receptors, or their binding—in this case, the binding of ACE2 to the spike protein. A similar result has been reported for *isoquercetin* [40]. A possible explanation for the pronounced virucidal effect is the use of different cellular receptors by the two viruses, with SARS-CoV-2 relying on ACE2 and HCoV-229E using aminopeptidase N (CD13) for cell entry. Furthermore, structural differences in the spike glycoprotein and viral proteases may lead to different sensitivities to the metabolites present in the extracts. We acknowledge that resolving the exact step-by-step mechanism of action is crucial for further development. In this study, we separated viral replication inhibition (post-entry antiviral effect) from direct extracellular virucidal action (virion inactivation). Our data show that the C3 extract exhibits both a potent post-infection replication inhibitory capacity against SARS-CoV-2 and ACV-resistant HSV-2, alongside a rapid direct virucidal effect within the first 5–15 min of contact. Our proposed pathways (e.g., 3CLpro/PLpro inhibition or modulation of NF-κB/MAPK cascades) remain well-supported hypotheses until these explicit combination trials are completed. HPLC-MS analysis revealed that both extracts contain a complex profile of phenolic acids and flavonols, which have been widely documented for their broad-spectrum antiviral efficacy. Among the identified phenolic compounds, chlorogenic acid and *dicaffeoylquinic acid* derivatives are known to play a role in blocking different steps of the viral replication cycle. Recent studies have shown that chlorogenic acid can effectively block the binding between the spike glycoprotein of SARS-CoV-2 and the ACE2 receptor of the host cell. Furthermore, *dicaffeoylquinic acids* exhibit strong binding affinity to viral structural proteins and enzymes, such as the main protease of coronaviruses, and have been shown to directly inhibit fusion, which is highly consistent with the pronounced virucidal effect observed for extract C3 in our study [43,44,45]. Regarding the glycosylated flavonols present in our extracts, *hyperoside (quercetin-3-O-galactoside*), *isoquercitrin (quercetin-3-O-glucoside*) and *kaempferol-3-O-galactoside* have been shown to target the catalytic pockets of highly conserved regions of viral proteases. Crucially, standard glycosylation alters lipophilicity, solubility and overall bioactivity compared to free aglycones. The higher antiviral activity of extract C3 (obtained from *C. campestris* parasitizing *Chenopodium* spp.), despite the lower content of free *quercetin* and *kaempferol* compared to C1, strongly supports the hypothesis of complex intramolecular interactions. Although free aglycones such as *quercetin* are potent inhibitors of intracellular replication, their presence together with high concentrations of glycosides (such as *hyperoside* and *isoquercitrin*) may induce antagonistic interactions, possibly due to noncovalent complexes or hydrogen bonds that limit the free fraction of active hydroxyl groups. This evidence from the literature supports our assumption that the optimal antiviral effect is determined by the specific ratio of flavonoid glycosides to free aglycones, and not just by the total amount of polyphenols [46,47].

### Limitations of This Study

A single-cycle test was not performed to determine the stage of the viral replication cycle at which the extracts act. This would, on the one hand, direct us more precisely to a viral target, and on the other hand, we could conduct molecular docking with greater accuracy to establish the capacity and strength of binding of individual metabolites to the target. The listed studies are planned to be conducted at a later stage. In this regard, all mechanisms of action listed in Section 3 are hypothetical, supported by the relevant citations. A limitation of this study is that MTT-based preservation of cell viability alone cannot establish the precise mechanism by which CPE is reduced. A limitation of this study is the lack of a control with Remdesivir.

## 4. Materials and Methods

### 4.1. Plant Extracts

In the present study, 6 aqueous and methanol extracts were used, obtained from stems and flowers of the plant species *Cuscuta epithymum* and *Cuscuta campestris* from the same habitat. Separate studies were conducted with methanol extracts from stems of *Cuscuta campestris* (C1) with host *Polygonum aviculare* and *Cuscuta campestris* (C3) with host *Chenopodium* spp. from the same habitat.

Methanol extracts—10 g of aerial parts (stem and/or flower) of frozen plant material were ground in liquid nitrogen and extracted with 15 mL of 100% methanol, followed by 20 min of vortexing at room temperature. After centrifugation for 10 min at 10,000 rpm, the supernatant was concentrated and dried in a vacuum concentrator at room temperature. The dry weight yield was 0.265 to 0.289 g. Before drying, 500 µL of the methanol extracts were filtered through a 0.45 micron filter membrane (CHROMAFIL PTFE, Chroma Ltd., Sofia, Bulgaria). The filtered sample was used for HPLC analysis.

For the aqueous extracts, 100 mg of plant material was ground with liquid nitrogen and 1 mL of water was added and vortexed for 30 min at room temperature. The centrifugation mode was the same as for the methanol extracts.

### 4.2. Cell Lines

Monolayer cell lines were used—MDBK, cells derived from bovine kidney (ATCC (No. CCL-22), American Type Culture Collection, Manassas, VA, USA), VERO E6 clone 76 (ATCC (No. CRL-1587)) and BGM—kidney epithelial cells from African green monkey (ECACC catalog no: 90092601, European Collection of Authenticated Cell Cultures, Salisbury, UK). The MDBK cell line was maintained in growth medium containing 8% FBS and maintenance medium with 4% serum, while VERO E6 and BGM cells were cultured in growth medium with 10% fetal calf serum and maintenance medium with 5% FBS, gentamycin 0.008 mg/mL, 2% NaHCO_3_ solution and 1% HCL solution for pH adjustment.

### 4.3. Viruses

The studies were conducted on the following viral models: human simplex virus type 1 (HSV-1) strain F, human simplex virus type 2 (HSV-2) strain DD (acyclovir-resistant), HCoV-229E (human coronavirus 229E) and SARS-CoV-2, 13th passage (isolated from a patient and had undergone 13 passages). The selection of HSV-1, HSV-2 and HCoV-229E was based on their clinical relevance and suitability, as representative models of various viral infections. HSV-1 and HSV-2 were selected as clinically important members of the Herpesviridae family, one of which is resistant. This allowed us to evaluate the antiviral activity of the extracts against two closely related herpesviruses. HCoV-229E was included as a representative human coronavirus training strain belonging to a different viral family, thus providing an additional model of an enveloped RNA virus with a different viral replication strategy. The selection of these viruses was also related to the availability of established and standardized experimental models in our laboratory.

### 4.4. Determination of Maximum Tolerable Concentration (MTC) and Cytotoxic Concentration 50 (CC50) Using MTT Assay

The maximum tolerable concentration (MTC) is the highest concentration of the test substance that does not cause damage or death of the treated cells. The cytotoxic concentration 50 (CC_50_) is defined as the concentration of the test substance at which 50% of the cells die as a result of the toxic effect of the substance.

MTC was determined at 72 h, both microscopically and by the MTT test [48]. The results were read spectrophotometrically at a wavelength of 540 nm with an ELISA reader Multiscan MX (Thermo Fisher Scientific, Sofia, Bulgaria). Cell survival was determined as the % of living cells treated with different concentrations of the test extract compared to untreated cell controls. The extracts were tested in concentration ranges from 1.25 mg/mL to 0.01 mg/mL.

The results obtained were graphically displayed by constructing a dose-response curve, from which the CC_50_ was determined.

### 4.5. Determining the Influence of the Tested Substances on Viral Replication Using MTT Test

To assess the antiviral activity of the tested extracts, the MTT test [48] was used and modified for rapid screening of compounds with potential antiviral activity [49,50,51].

The assessment of the preserved cell viability in infected and substance-treated cells serves as an indicator of the presence and degree of antiviral activity of the tested compounds.

When the cell monolayer reached 90–100% confluence in each well of a 96-well plate, the coverslip was removed, and the cells were infected with virus in a volume of 0.1 mL per well at a working dose of 100 TCID_50_. After adsorption of the virus for 1 h at 37 °C, 0.1 mL of DMEM maintenance medium without dissolved substance was added to the control wells (at least 3). The remaining wells were added with the same volume of medium, but with substance at the corresponding concentrations. Each of the concentrations was added to three wells. The substances at their MIC and several two-fold dilutions below it were used in the experiments. The following protocol was applied:

- Cell control—uninfected and untreated cells.
- Viral control—cells infected with the specified working dose of virus, but not treated with the extract.
- Samples—treated cells (infected and treated with substance), with at least three wells used for each concentration.

To prevent false readings from extract turbidity, color or direct chemical reduction of the tetrazolium salt, we systematically included extract-only controls (uninfected, extract-treated in MTC cell controls) that were used as background during spectrophotometric measurement at 540 nm.

After reporting the results, the activity of the tested substance was expressed as % protection of the infected cells and was determined by the following formula:

[ODpr − ODcv]/[ODccl. − OD cv] × 100 (%)(1)

ODpr—absorbance of infected and substance-treated cells;
ODcv—absorbance of virus-infected cells (virus control without substance);
ODccl.—absorbance of uninfected and substance-treated cells (cell control).

Each experiment was repeated 3 to 5 times, and each sample or control was plated in at least three wells. Standard statistical processing was used, and the *p* value was <0.05.

### 4.6. Testing for Antiviral Activity by Neutral Red Staining

Neutral red is a dye that accumulates in the lysosomes of living cells by active endocytosis. The amount of accumulated neutral red is proportional to the number of viable cells in the culture. After culturing the cells for 72 h in the presence of different concentrations of the tested methanol extracts, the medium was removed and replaced with 0.1 mL of serum-free medium containing neutral red (1 mL of 0.4% neutral red solution in 80 mL of medium). The cells were incubated for 3 h in a thermostat at a temperature of 37 °C and 5% CO_2_. After incubation, the medium was removed, and each well was washed with 0.2 mL of PBS, after which 0.1 mL of an ex tempore destabilizing solution containing 96% ethyl alcohol, distilled water and acetic acid in a ratio of 50:49:1 (vol:vol:vol) was added. The plates were incubated for 10–15 min, and the absorbance was measured at 540 nm on an ELISA reader Multiscan MX.

### 4.7. Antiviral Activity Assay Using Plaque-Forming Method

The VERO E6 cell line was used. The antiviral activity of methanol extracts from the plant *Cuscuta campestris* was evaluated using a viral model SARS-CoV-2. The solutions of substances C1 and C3 were at the MTC and at several lower concentrations. The protocol used was according to the method of Dulbecco et al. [52].

Antiviral activity was assessed by determining the viral titer in the samples relative to the control, in plaque-forming units (PFU/mL). Samples with no visible plaques were considered below the limit of detection (LOD) of the assay, which was defined as 5 PFU/mL based on the minimum detectable 1 PFU in a 0.2 mL inoculum volume.

### 4.8. Effect of C3 Extract on Extracellular Virions

The effect on extracellular virions was determined by the direct contact method. Equal volumes of undiluted virus suspension and the test extract at the MNC were mixed and incubated for different time intervals (5, 15, 30, 60, 120 and 240 min) at 37 °C. Viral controls contained undiluted virus suspension and maintenance medium without solute in it and were incubated for the same intervals together with the samples. At the end of each time interval, the samples and controls were frozen and thawed. Their titers were then determined by the method of Reed and Muench [33].

### 4.9. Flavonoid Profile of C. campestris and C. epithymum Extracts

The extraction and determination of the polyphenol profile of the extracts were performed according to the protocol described in our previous studies [30].

### 4.10. Flavonoid Profile of C1 and C3

HPLC analyses were performed on the Agilent Technolgies 1260 Infinity II LC system (Agilent Technolgies, Inc., Santa Clara, CA, USA) including a quaternary pump, autosampler, multicolumn thermostat, WR Diode Array Detector (DAD), and a Quadrupole Time-Of-Flight (QTOF) Agilent 6546 detector. ESI-MS spectra were recorded in negative ion ([M-H]^−^) mode in the interval between *m*/*z* 50 and 1500 and 120 V voltage of the fragmentor. Compounds were separated on a Knauer Eurospher II 100-2 C18 column (KNAUER Wissenschaftliche Geräte GmbH, Marvel Ltd. (Marvel), Plovdiv, Bulgaria), 150 × 2 mm, 2 μm particle size. The temperature of the column was 28 °C. The mobile phase consisted of 0.1% formic acid in water (A) and 0.1% formic acid in acetonitrile (B). The gradient at a flow rate of 0.2 mL min−1 was 0–40 min from 0 to 46% B; 40–42 min 100% B; 42–52 min 100% B isocratic; and 52–54 min to 0% B. UV spectra were measured at 190–500 nm.

## 5. Conclusions

The 13 identified compounds varied statistically significantly between the two species *C. campestris* and *C. epithymum*, including flavone, *chlorogenic acid*, *hyperoside*, *isoquercitrin*, *kaempferol-3-O-galactoside*, *quercetin*, *kaempferol-3,7-O-diglucoside* and *dicaffeoylquinic acid*. The total polyphenol content was higher in *C. epithymum* compared to *C. campestris*. The fact that the total polyphenol fraction in *C. epithymum* was higher does not necessarily mean higher antiviral activity. We consider the highest antiviral activity against the resistant strain of HSV 2 (DD) established upon treatment with the methanol extract of the stem of *C. campestris*, characterized by the lowest IC_50_ value (0.01 mg/mL) and the highest calculated selectivity index (SI = 28), as a particularly valuable result. Of the two methanol extracts of *C. campestris* obtained from plants with different hosts, better antiviral activity was shown by extract C3, with host Chenopodium spp, with an IC_50_ of 0.03 mg/mL, a higher selectivity index (SI = 12) and a viral inhibition rate of about 100% against SARS-CoV-2 when the extract was applied at the MTC. Another important result, in our opinion, is that, depending on the host plant, the polyphenolic composition of the extracts and the amount of secondary metabolites differ. It was also found that the MTT assay was not suitable for determining cell viability in SARS-CoV-2-infected VERO E6 cells, as the values reported for viral and cell control were the same, with a clear CPE in infected and untreated wells. This result could hypothetically mean that cells infected with this virus have partially preserved mitochondrial activity.

## Figures and Tables

**Table 1:** Identified compounds in C1 and C3 after HPLC/MS analysis. ↓, decrease; ↑, increase.

Peak	tR (min)	Compound	Trivial Name	UV λ Max (nm)	[M-H]^−^	CC1 vs. CC3 (Fold Change)
1	18.1	Chlorogenic acid		218, 300, 325	353.086	0.97 ↓
2	21.6	Kaempferol-3,7-O-diglucoside		265, 345	609.1435	1.85 ↑
3	26.4	Quercetin-3-O-galactoside	Hyperoside	255, 355	463.0868	1.03 ↑
4	26.8	Quercetin-3-O-glucoside	Isoquercitrin	255, 355	463.0868	1.03 ↑
5	28.1	Kaempferol-3-O-galactoside		265, 343	449.0922	1.50 ↑
6	28.8	Kaempferol-3-O-glucoside	Astragalin	265, 350	447.0914	1.28 ↑
7	29.0	Isorhamnetin-7-glucoside		255, 353	447.0911	1.34 ↑
8	29.4	Isorhamnetin-3-O-glucoside		255, 345	447.1025	1.54 ↑
9	34.4	Quercetin		255, 370	301.0347	4.29 ↑
10	37.4	Kaempferol-3-O-β-(6"-O-trans-p-coumaroyl)-glucopyranoside	Tiliroside	268, 315	593.1279	1.75 ↑
11	38.5	Kaempferol		265, 366	285.0389	6.84 ↑
12	39.2	Isorhamnetin		256, 368	315.0503	6.91 ↑

**Table 2:** Toxicity and antiviral activity of extracts from *C. campestris* and *C. epithymum*.

	Cell Viability (Cytotoxicity)		Antiviral Activity HSV-1 (F)			Antiviral Activity HSV-2 (DD)		
Extracts	MTC (mg/mL)	CC_50_ (mg/mL)	Cell Protection (%) When Extracts Are Added in MTC	IC_50_	SI = CC_50_/IC_50_	Cell Protection (%) When Extracts Are Added in MTC	IC_50_	SI = CC_50_/IC_50_
C ep. stem, MetOH	0.16	0.36	89.5	0.021	17	80	0.116	3.1
C. camp stem, MetOH	0.16	0.28	96.1	0.023	12	99.8	0.01	28
C. camp. flower, MetOH	0.16	0.42	96.9	0.027	15.5	98.2	0.02	21
C. ep stem, H_2_O	0.16	0.42	83.1	0.038	11	73.4	0.04	10.5
C. camp stem, H_2_O	0.16	0.68	71.2	0.038	17.8	70	0.067	10
C. camp flower, H_2_O	0.16	0.52	80.4	0.038	13.6	73	0.061	8.5

Legend: MTC—maximum tolerable concentration; CC_50_—cytotoxic concentration that reduces cell viability by 50%; IC_50_—inhibitory concentration that reduces viral activity by 50%; SI—selectivity index (CC_50_/IC_50_); HSV-1—herpes simplex virus type 1; HSV-2—herpes simplex virus type 2; F—HSV-1 strain F; DD—HSV-2 strain DD; MetOH—methanol; H_2_O—water; C. ep.—*C. epithymum*; C. camp.—*C. campestris*. Cell protection is expressed as a percentage (%). Concentrations are expressed in mg/mL. Each experiment was repeated at least 3 times to ensure reproducibility of the results. At least three wells were treated with each extract.

**Table 3:** Cytotoxicity of C1 and C3 for cell lines—MDBK, VERO E6 and BGM.

	Cell Viability (Cytotoxicity)			
	C1 Extract		C3 Extract	
Cell Lines	MTC (mg/mL)	CC_50_ (mg/mL)	MTC (mg/mL)	CC_50_ (mg/mL)
MDBK	0.16	0.431	0.16	0.365
VERO E6	0.16	0.427	0.16	0.296

Legend: MTC—maximum tolerable concentration; CC_50_—cytotoxic concentration that reduces cell viability by 50%. Concentrations are expressed in mg/mL.

**Table 4:** Antiviral activity of C1 and C3 against HSV-2 strain DD.

	Cell Viability (Cytotoxicity)		Antiviral Activity HSV-2 (DD)		
	MTC (mg/mL)	CC_50_	Protection (%) When Extracts Are Added in MTC	IC_50_	SI = CC_50_/IC_50_
C1	0.16	0.365	66.3	0.05	7.3
C3	0.16	0.365	100	0.03	12

Legend: MTC—maximum tolerable concentration; CC_50_—cytotoxic concentration that reduces cell viability by 50%; IC_50_—inhibitory concentration that reduces viral activity by 50%; SI—selectivity index (CC_50_/IC_50_); HSV-2—herpes simplex virus type 2; DD—HSV-2 strain DD. Cell protection is expressed as a percentage (%). Concentrations are expressed in mg/mL.

**Table 5:** Antiviral effect of extracts C1 and C3 determined by the plaque formation method.

Extracts	Concentration (mg/mL)	Virus Titer (PFU/mL)	Relative Viral Infectivity (%)	Plaque Reduction (%)
C1	0.16	Below the limit of detection (<5PFU/mL)	<0.01	~100.0
	0.08	1.44 × 10^6^	94.1	5.9
	0.04	1.42 × 10^6^	92.8	7.2
	0.02	1.4 × 10^6^	91.5	8.5
	Control virus	1.53 × 10^6^	100.0	0.0
C3	0.16	Below the limit of detection (<5 PFU/mL)	<0.01	~100.0
	0.08	Below the limit of detection (<5 PFU/mL)	<0.01	~100.0
	0.04	1.33 × 10^6^	74.3	25.7
	0.02	1.48 × 10^6^	82.7	17.3
	Control virus	1.79 × 10^6^	100.0	0.0

The presented results show that the C3 extract inhibits plaque formation to a greater extent, even at lower concentrations. The values of 0 PFU/mL were below the limit of detection (LOD) of the assay. The LOD was defined as 5 PFU/mL, calculated from a minimum detection threshold of 1 PFU per 0.2 mL of inoculum volume. Relative viral infectivity for these samples was calculated based on the LOD value.

**Table 6:** Virucidal effect of C3 on the extracellular forms of SARS-CoV-2 and HSV-2 strain DD.

SARS-CoV-2				HSV-2 (DD)		
Time Interval (min)	Titer of Control Virus	Titer of Treated Virus	∆lg	Titer of Control Virus	Titer of Treated Virus	∆lg
5	5.5	3	2.5	5.67	4.33	1.34
15	5.5	3.5	2	5.67	4.33	1.34
30	5	3.5	1.5	5.67	5.67	
60	4.67	3.5	1.17	4.5	4.5	
120	4.67	3.5	1.17	4.5	4.5	
240	4.67	4	0.67	4.5	4.5	

## Data Availability

The original contributions presented in this study are included in the article/Supplementary Materials. Further inquiries can be directed to the corresponding author.

## Supplementary Materials

The following supporting information can be downloaded at: https://www.mdpi.com/article/10.3390/ijms27188120/s1.

## Abbreviations

The following abbreviations are used in this manuscript:

MTC	Maximum Tolerable Concentration
HSV-1	Human simplex virus type 1
HSV-2	Human simplex virus type 2
HCoV-229E	Human coronavirus 229E
