# Supplementary material for: Evaluation of the Antiviral Activity of Cuscuta Extracts Against HSV-1, HSV-2 (ACV-Resistant Strain), HCoV229E and SARS-CoV-2
Source: Int J Mol Sci. 2026 Sep 12;27(18):8120. doi: 10.3390/ijms27188120 (PMC13607513; doi:10.3390/ijms27188120)
Supplement: Supplementary file 1 [file ijms-27-08120-s001.zip › ijms-4518986-supplementary.pdf]

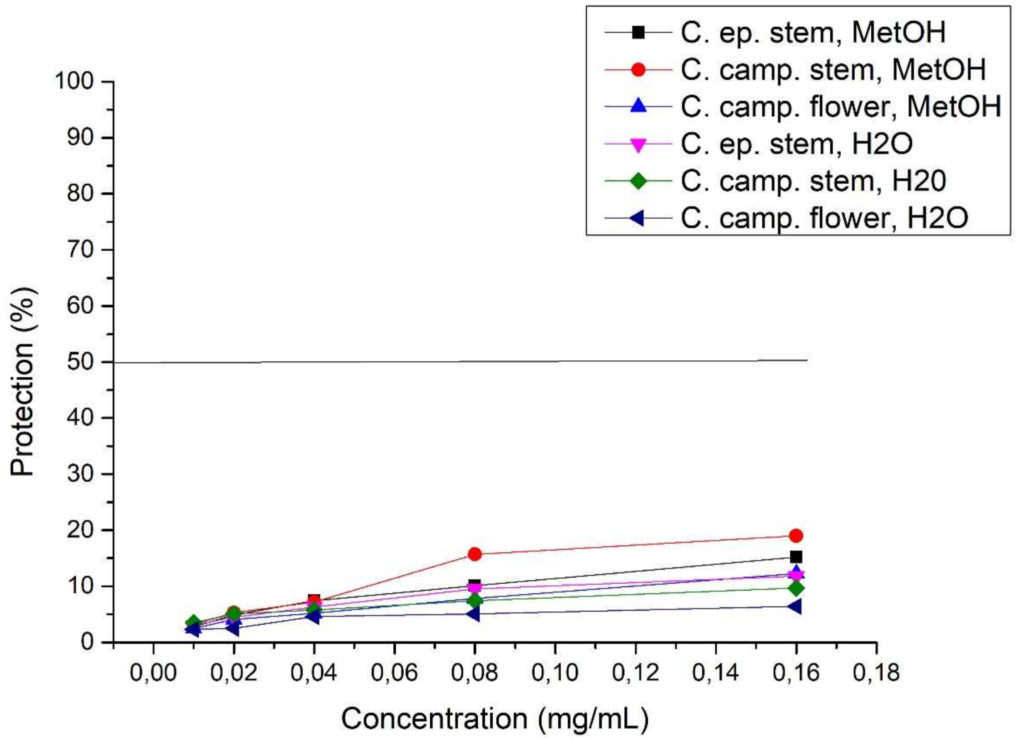

Supplementary Figure S1. Antiviral activity of *C. campestris* and *C. epithymum* against HCoV229E. All tested aqueous and methanolic extracts showed low levels of protection against HCoV229E, with none reaching the 50% protection threshold at any of the tested concentrations. The highest percentage of protection against HCoV229E was observed for the methanolic stem extract of *C. campestris* at the MTC (0.16 mg/mL).

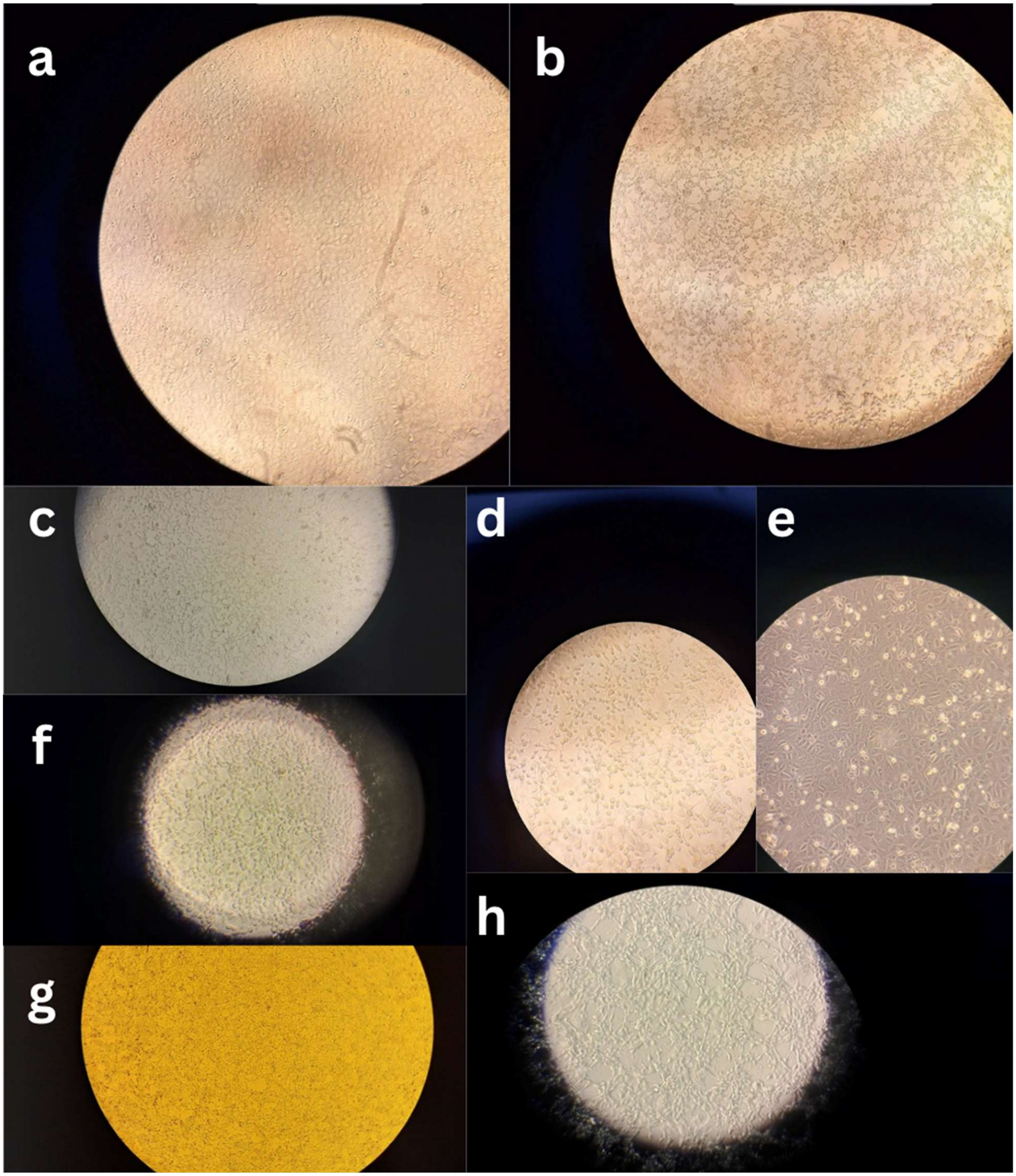

Supplementary Figure S2. (**a**) virus infected cells and treated with C3-0.16mg/mL-48h; (**b**) virus control at 48h-cells infected with virus but not treated with extract; (**c**) virus infected cells and treated with C3-0.16mg/mL-48h; (**d**) virus control at 72h-cells infected with virus but not treated with extract; (**e**) control cells-cells not infected with virus and not treated with substance; (**f**) virus infected cells and treated with C3-0.08mg/mL-48h-high magnification; (**g**) virus infected cells and treated with C3-0.08mg/mL-48h-low magnification of the microscope; (**h**) virus infected cells and treated with C3-0.02mg/mL-48h. The remaining dilutions of the extract show a picture like the virus control.
